# Quantitative Analysis of Changes to Meibomian Gland Morphology Due to S-1 Chemotherapy

**DOI:** 10.1167/tvst.7.6.37

**Published:** 2018-12-28

**Authors:** Kazuyoshi Ohtomo, Reiko Arita, Rika Shirakawa, Tomohiko Usui, Hiroharu Yamashita, Yasuyuki Seto, Satoru Yamagami, for the TS-1 Multicenter Study Group

**Affiliations:** 1 Department of Ophthalmology, Japan Community Healthcare Organization, Tokyo Shinjuku Medical Center, Tokyo, Japan; 2 Department of Ophthalmology, University of Tokyo Hospital, Tokyo, Japan; 3 Department of Ophthalmology, Itoh Clinic, Saitama, Japan; 4 Department of Gastrointestinal Surgery, Graduate School of Medicine, University of Tokyo, Tokyo, Japan; 5 Department of Ophthalmology, Nihon University Itabashi Hospital, Tokyo, Japan

**Keywords:** meibography, meibomian gland, meibomian gland area ratio, S-1, tegafur

## Abstract

**Purpose:**

The aim of this prospective, noncomparative, observational case series was to evaluate changes to meibomian gland morphology in patients undergoing S-1 chemotherapy with the use of noninvasive meibography and analytical software.

**Methods:**

Patients aged ≥20 years and undergoing S-1 chemotherapy were included. Ophthalmologic examinations were performed before S-1 administration (Pre) and at 1, 3, and 6 months afterward (1M, 3M, and 6M, respectively). Meibography images were analyzed using analytical software. The ratio of the total meibomian gland area relative to the whole measurement area (meibomian gland area ratio [MGAR]) and the rate of change to the MGAR (MGAR at Pre, 1M, 3M, or 6M)/(MGAR at Pre) × 100 were calculated.

**Results:**

In total, 28 eyelids of seven men (mean age, 68.9 ± 6.3 years) were studied. The mean MGAR of the upper and lower eyelids significantly decreased at 3M (*P* = 0.0246 and *P* = 0.00892, respectively) and 6M (*P* < 0.0001 and *P* < 0.0001, respectively). There was a significant negative correlation between the cumulative dose of S-1 and the rate of change to the MGAR of the upper (*P* < 0.0001, *r* = −0.77) and lower (*P* < 0.0001, *r* = −0.785) eyelids. However, there was no other significant difference.

**Conclusions:**

The meibomian gland area decreased after S-1 administration with significant correlations between the rate of change to the MGAR and the mean cumulative dose of S-1.

**Translational Relevance:**

Systemic S-1 administration decreased the MGAR in a dose-dependent manner; thus, clinicians should pay more attention to morphologic changes to the meibomian glands during early treatment with S-1.

## Introduction

The combination drug S-1 (tegafur, gimeracil, and oteracil potassium) is an oral anticancer agent for treatment of malignant tumors^1^ of the stomach,^2^ pancreas,^3^ colon,^4^ and breast.^5^ Tegafur is a prodrug of 5-fluorouracil (5-FU) with anticancer effects. Gimeracil inhibits 5-FU degradation and extends the half-life of 5-FU in blood. Oteracil potassium is a phosphorylation inhibitor that blocks 5-FU generation in the gastrointestinal tract, where otherwise higher levels would result in higher toxicity. 5-FU is metabolized and decomposed by the same pathway and the same enzyme as uracil in vivo. Metabolism of 5-FU in the liver produces two active metabolites: fluorouridine triphosphate, which disrupts RNA function, and fluorodeoxyuridine monophosphate, which inhibits DNA synthesis, resulting in anticancerous effects.^6^ However, these effects on RNA and DNA are not only restricted to cancerous cells, but also affect normal cells, leading to side effects throughout the body. The ophthalmic adverse effects of S-1 include corneal disorders,^7–9^ conjunctival disorders,^10^ lacrimal duct obstruction,^8–12^ and meibomian glands dysfunction.^7,8^ Keratoconjunctival disorders lead to photophobia, irritation, and decreased visual acuity, while lacrimal duct obstruction is characterized by epiphora and discharge. The meibomian glands play an important role in protecting the ocular surface, and 86% of all cases of dry eyes occur due to meibomian gland dysfunction.^13^ The ocular tear film consists of a lipid and an aqueous layer, and the meibomian glands secrete the lipid layer and prevent excessive evaporation of the tear film.

Since ocular symptoms, tear-film–related parameters, changes to the shape of the meibomian glands over time, and the quantitative relationship between S-1 chemotherapy and meibomian gland dysfunction have not been fully investigated, the aim of the present study was to evaluate the effects of S-1 chemotherapy on the ocular surface, including morphologic changes to the meibomian glands, with the use of noninvasive meibography^14^ and analytical software (Meboscore Analysis; Topcon Corporation, Tokyo, Japan).^15^

## Methods

### Subjects

This prospective case series was approved by the Ethics Committee of the University of Tokyo Hospital and was conducted in accordance with the tenets of the Declaration of Helsinki. The enrollment period spanned 2 years between January 2015 and December 2016, and follow-ups were carried out for up to 1 year. S-1 monotherapy was used as an adjuvant for all patients with gastric cancer after surgery, and chemotherapy was administered for 1 year. Before S-1 administration, the patients were referred from the Department of Gastrointestinal Surgery to the Department of Ophthalmology of the University of Tokyo Hospital. All patients provided written informed consent after a thorough explanation of the present study was given by an ophthalmologist (OK). The study cohort was limited to patients aged ≥20 years and treated with S-1 as an initial anticancer drug. All patients presenting with active keratoconjunctivitis or lacrimal duct obstruction were excluded.

In total, 44 eyelids of 11 patients were enrolled for the study. A total of four patients were excluded (two who discontinued S-1 therapy because of systemic side effects and two who dropped out shortly after the study had begun). In the end, seven patients (28 eyelids) completed the 6-month follow-up.

Ophthalmologic examinations were performed during each visit before S-1 administration (Pre) and at 1, 3, and 6 months afterward (1M, 3M, and 6M, respectively). The examinations included a medical interview to record subjective complaints, anterior-segment optical coherence tomography to measure tear meniscus height (TMH), a slit lamp examination, which included fluorescein staining for detection of keratoconjunctival disorders, tear film break-up time (BUT) measurements, in addition to meibography, and an irrigation test for lacrimal passage patency. The best corrected visual acuity (BCVA) was evaluated at Pre, 3M, and 6M. BCVA scores were logarithmically converted into the minimum angle of resolution (logMAR) values. The administration dose of S-1 was recorded at each visit in the medical records maintained by the Department of Gastrointestinal Surgery.

The medical interview included questions to assess the presence of epiphora, discharge, photophobia, irritation, and decreased visual acuity. The TMH was obtained with a Fourier-domain swept-source anterior-segment optical coherence tomography apparatus (SS-1000; Tomey Corporation, Nagoya, Japan).^16^ Fluorescein staining was conducted using a blue-free filter. The total corneal fluorescein score was the sum of 0–3 points assigned to each of the nasal, temporal, central, superior, and inferior areas (range, 0–15 points).^17^ The conjunctival fluorescein score was also the sum of 0–3 points assigned to the nasal and temporal areas (range, 0–6 points).^18^ After fluorescein staining, the BUT was measured starting from eye opening and until break up. In case of blinking, the time to blink was measured as the BUT. The average BUT was obtained from three measurements and recorded. The upper and lower eyelids were everted, and images of the meibomian gland were obtained by noninvasive meibography^14^ and analyzed with analytical software.^15^ The ratio of the total area occupied by meibomian glands relative to the total analysis area (meibomian gland area ratio [MGAR]) was calculated in all patients. The rate of change to the MGAR was calculated according to the following formula: (MGAR at Pre, 1M, 3M, or 6M)/(MGAR at Pre) × 100. All patients underwent the irrigation test for lacrimal duct patency from the upper and lower lacrimal canaliculus with a Bangerter lacrimal probe cannula. When there were epiphora symptoms, lacrimal duct obstruction was identified by regurgitation from the upper and lower puncta during the irrigation test.

### Statistical Analysis

Statistical analyses were performed using software (EZR; Saitama Medical Center, Jichi Medical University, Saitama, Japan),^19^ which is a graphical user interface for R (The R Foundation for Statistical Computing, Vienna, Austria). Calculation power was calibrated using the post hoc test. At α = 0.05 and calculation power = 0.80, the required sample size was 12 eyelids. Thus, the sample size had sufficient calculation power. One-way analysis of variance was used for normally distributed variables, and the Kruskal-Wallis test was used when those assumptions were not met to compare the values of BCVA logMAR, TMH, fluorescein score, BUT, and MGAR at Pre, 1M, 3M, and 6M. Dunnett's test was used for multiple comparisons. The rate of change to the MGAR was calculated according to the following formula: (MGAR at Pre, 1M, 3M, or 6M)/(MGAR at Pre) × 100. The nonparametric Spearman's rank correlation coefficient was used to identify any correlation between the cumulative S-1 dose and the variables of the logMAR value of BCVA, TMH, fluorescein scores of the cornea and conjunctiva, BUT value, and the rate of change to the MGAR in the upper/lower eyelids. A probability (*P*) value of <0.05 was considered statistically significant.

## Results

Table 1 shows the clinical characteristics of the seven patients (28 eyelids). The mean age of the patients was 68.9 ± 6.3 (range, 61–78) years. All patients were men. The mean estimated glomerular filtration rate was 69.4 ± 12.4 (range, 50.6–85.8) mL/min/1.73 m^2^.

**Table 1:**
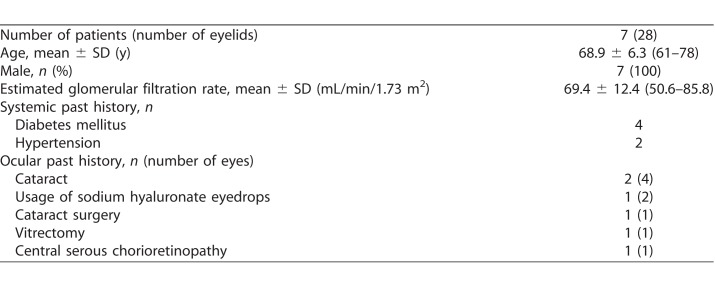
Clinical Characteristic of Patients in the Present Study

Epiphora was present in 0%, 14%, 14%, and 43%, and discharge was present in 0%, 0%, 14%, and 43% of the patients at Pre, 1M, 3M, and 6M, respectively. Photophobia developed only in 29% of the patients at 6M. Symptoms of irritation were reported by 0%, 14%, 14%, and 29% of the patients at Pre, 1M, 3M, and 6M, respectively. However, none complained of decreased visual acuity during the study period.

There was a systemic past history of diabetes mellitus in four patients and of hypertension in two. Past ocular histories included cataracts in four eyes of two patients, use of sodium hyaluronate eyedrops in two eyes of one patient, cataract surgery in one eye of one patient, vitrectomy in one eye of one patient, and central serous chorioretinopathy in one eye of one patient.

**Figure 1:**
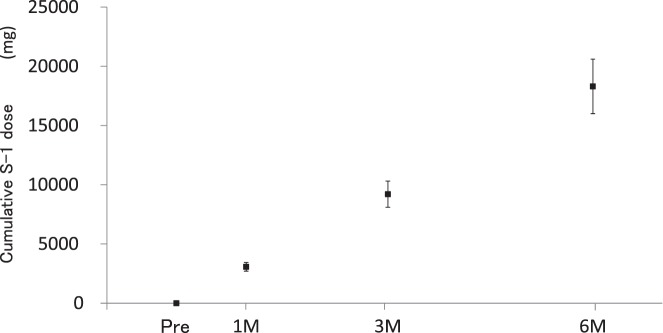
Cumulative S-1 dose. Cumulative S-1 dose increases with time. Black squares mean cumulative dose of S-1. Bars: standard deviation.

The mean cumulative S-1 dose increased with time (Fig. 1): 3069 ± 368, 9206 ± 1103, and 18,309 ± 2301 mg at 1M, 3M, and 6M, respectively.

**Figure 2:**
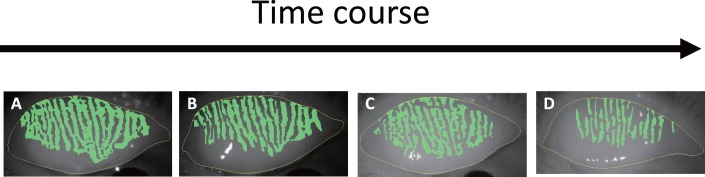
Representative meibomian gland images of a right upper eyelid of a 61-year-old male before and after S-1 administration. (A) Pre, (B) 1M, (C) 3M, and (D) 6M. Meibomian gland images were clearly visualized before and after S-1 administration on meibography. With time, the meibomian gland area that was colored green visibly decreased.

Figure 2 shows evidence of morphologic changes to the meibomian gland of an upper eyelid due to S-1 chemotherapy. Meibographic images of the meibomian gland of patients were clearly visualized before and after S-1 administration. With time, the meibomian gland area that was colored green decreased visibly. In the case shown here, the MGARs were 41%, 32%, 31%, and 14.0% at Pre, 1M, 3M, and 6M, respectively. Additionally, the rate of change to the MGAR was 100%, 77%, 75%, and 34% at Pre, 1M, 3M, and 6M, respectively.

**Figure 3:**
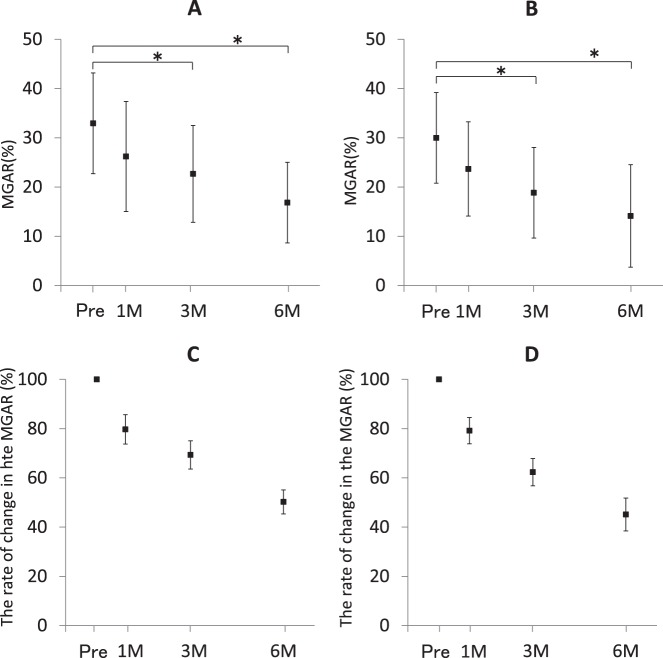
Change in the MGAR on (A) upper and (B) lower eyelids, and the rate of change in the MGAR on (C) upper and (D) lower eyelids. (A) There were significant differences in MGAR reduction in the upper eyelids at 3M and 6M (P = 0.0246 and P < 0.0001, respectively). (B) There were also significant differences in MGAR reduction in the lower eyelids at 3M and 6M (P = 0.00892 and P < 0.0001, respectively). (A, B) Asterisks show significance. (A, B) Black squares mean value of the MGAR. (A, B) Bars: standard deviation. (C, D) The rate of change in the MGAR was calculated by using the following formula: (MGAR at Pre, 1M, 3M, or 6M)/(MGAR at Pre) × 100. (C) The percent change from the baseline value in the upper eyelids decreases to 50% at 6M. (D) The percent change from the baseline value in the lower eyelids also decreases to 45% at 6M. (C, D) Black squares mean value of the rate of change in the MGAR. (C, D) Bars: standard error.

There were significant differences in MGAR reduction of the upper eyelids at 3M and 6M (*P* = 0.0246 at 3M and *P* < 0.0001 at 6M, Fig. 3A). The mean MGAR and percent change from the baseline value were 33% and 100% at Pre, 26% and 79% at 1M, 23% and 69% at 3M, and 17% and 50% at 6M, respectively (Figs. 3A and 3C). There were also significant differences in MGAR reduction of the lower eyelids at 3M and 6M (*P* = 0.00892 and *P* = <0.0001, respectively, Fig. 3B). The mean MGAR and percent change from the baseline value were 30% and 100% at Pre, 23% and 78% at 1M, 19% and 62% at 3M, and 14% and 45% at 6M, respectively (Figs. 3B and 3D).

**Figure 4:**
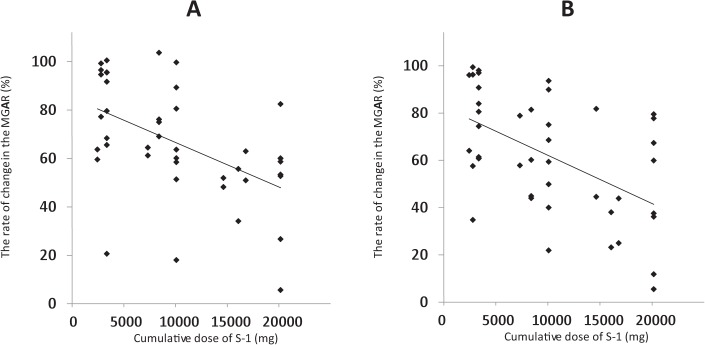
Correlation between the rate of change in the MGAR in the upper and lower eyelids and the mean cumulative dose of S-1. There was a significant negative correlation between the rate of change in the MGAR in the (A) upper and (B) lower eyelids and the mean cumulative dose of S-1 (P < 0.0001, r = −0.77 and P < 0.0001, r = −0.785, respectively).

There was a significant negative correlation between the rate of change to the MGAR of the upper and lower eyelids and the mean cumulative dose of S-1 (*P* < 0.0001, *r* = −0.77 and *P* < 0.0001, *r* = −0.785, respectively; Figs. 4A and 4B).

Table 2 shows the temporal changes of each variable. There was a significant difference in the logMAR values of BCVA between Pre and 6M (*P* = 0.0105). However, there was no significant difference in the TMH, fluorescein scores of cornea or conjunctiva, or BUT values. There were no patients with lacrimal duct obstruction during the study period. The mean logMAR BCVA value was correlated to the mean cumulative S-1 dose (*P* = 0.0222, *r* = 0.352). There were no other correlations among the variables of TMH, fluorescein score of cornea and conjunctiva, or BUT values with the cumulative S-1 dose (data not shown).

**Table 2:**
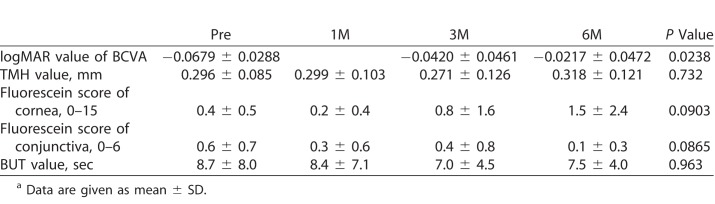
Temporal Changes on Each Parameter^a^

## Discussion

To our knowledge, this is the first study to evaluate morphologic changes to the meibomian glands over time and the correlation between the rate of change to the MGAR of the upper and lower eyelids with the mean cumulative S-1 dose. There was a significant negative correlation between the rate of change to the MGAR of the upper and lower eyelids and the mean cumulative S-1 dose.

The results of this study did not confirm differences between cases with and without the use of S-1. However, a previous study^7^ reported differences in meibomian grading and the morphology of meibomian glands between the healthy control group and the S-1 group, suggesting that S-1 may cause meibomian gland injury. Generally, morphologic changes are caused by aging, the use of contact lenses, allergic conjunctivitis, and the use of glaucoma eyedrops.^20^ Despite deterioration due to aging, morphologic changes occurred on a monthly basis in response to the use of S-1. Thus, time was not a confounding factor. Moreover, to exclude other confounding factors, the study cohort was limited to patients who received S-1 monotherapy after gastric cancer surgery. Therefore, the results represent only the side effects of S-1 administration. In brief, these findings suggest that systemic S-1 administration decreases the MGAR in a dose-dependent manner.

Mizoguchi et al.^8^ evaluated meibomian gland disorders using the meiboscore among patients administered S-1. The meiboscore assigns values to meibomian gland loss from 0 to 3 points (with 0 meaning no loss) and is useful to evaluate the shape of the meibomian glands.^14^ Another article^7^ defined meibomian gland disorders when the remaining meibomian glands declined to the central two-third of the tarsus in patients who were administered S-1. As the MGAR is correlated with the meiboscore,^15^ the MGAR was used in the present study to evaluate the remaining meibomian gland area. As previously explained, the MGAR is the ratio of the total meibomian gland area relative to the whole measurement area. Therefore, the MGAR not only ensures a quantitative evaluation, but also allows for temporal change in the same individual to be sensitively evaluated, as confirmed by the results of the present study.

The difference in the MGAR reduction between upper and lower eyelids was not compared in the present study. Anatomically, the upper eyelid is wider than the lower eyelid. Furthermore, it is necessary to consider the influence of gravity and blood flow from the viewpoint of localization. Therefore, no comparison could be made between the upper and lower eyelids.

5-FU (metabolized from tegafur) contained in the tear fluid^8^ and blood^12^ may cause tissue damage, which may explain the drastic effect on the meibomian glands, as compared with that on the keratoconjunctiva. Because of the abundant blood flow in the eyelid compared to that in the conjunctiva, it is conceivable that the meibomian glands are more exposed to 5-FU than the keratoconjunctiva. Furthermore, although the fluorescein score of the cornea decreased, this decrease was not significant (Table 2). The disappearance of the meibomian glands was observed with the development of corneal disorders.^8,9^ Because the meibomian glands protect the ocular surface and cornea,^20^ we speculated that the deterioration of cornea became apparent only after the meibomian glands began to disappear.

There have been no reports on risk factors of meibomian gland disorders and the relationship between the total S-1 dose and disappearance of the meibomian glands. Our results showed that the meibomian gland area decreased with cumulative S-1 doses.

Other ocular complications due to S-1 include lacrimal duct obstruction,^8–12^ corneal abnormalities,^7,8,11^ and conjunctival disorders.^10^

Lacrimal duct obstruction of the canaliculus develops in 8%–16% of patients administered S-1,^9,11,12^ which is believed to be caused by S-1–induced inflammation of the lacrimal duct, leading to lacrimal obstruction due to tissue fibrosis.^8,11,12^ In the present study, although symptoms of epiphora and discharge (indicating probable lacrimal duct obstruction) increased over time, no lacrimal duct obstruction was observed and there were no significant changes to TMH for 6 months. These symptoms may imply potential inflammation occurring on the ocular surface due to S-1 administration, and this paradoxical divergence between the symptoms and findings may mean that the tear volume on the ocular surface is adjusted by the maintained lacrimal drainage function.^21^ As the administration period and dose of 5-FU are related to lacrimal duct obstruction,^22^ we recommend close follow-up of patients administered S-1 for more than 6 months.

A study comparing the corneas of normal subjects to those of patients who were administered S-1 (matched by age and gender) found worse corneal staining and BUT scores (average duration of administration, 21.8 months; total cumulative dose, 41 g).^7^ The reason we did not find a decrease in corneal staining or BUT scores in our study seems to be the difference in the total cumulative dose and duration of administration.

To our knowledge, no study has yet reported conjunctival findings of fluorescein staining among patients administered S-1. However, conjunctivitis associated with S-1 administration^10^ is thought to be due to a complex disorder including drug-induced impairment, corneal disorders, meibomian gland dysfunction, and lacrimal obstruction.

With respect to the decrease in visual acuity at 6M, the fluorescein score of the cornea decreased, but this decrease was not significant (Table 2). It was thought that the cornea was hindered due to meibomian gland dysfunction and/or the side effects of S-1, which caused a mild decrease in logMAR visual acuity. Moreover, given the short 6-month follow-up period, it may be necessary to study patients for longer periods.

There were some limitations to this study that should be addressed. First, this study was conducted in a single hospital, and the sample size was small. Because all patients were administered S-1 for gastric cancer after surgery, it was difficult to compare our patients to controls receiving a placebo. Second, the follow-up period after S-1 administration was only 6 months. Third, although the function of the meibomian glands by observation of the orifices and surroundings areas was not conducted in the current study, according to a previous study,^8^ tegafur induces morphologic changes to the meibomian glands because 5-FU metabolism by tegafur causes inflammation of the meibomian glands, resulting in keratinization and fibrosis, followed by obstruction of the orifices, and finally disappearance of the glands.

In conclusion, we demonstrated morphologic changes to the meibomian glands in patients administered S-1 for 6 months. Meibomian gland areas decreased after the administration of the chemotherapeutic agent, and there were significant correlations between the rate of change to the MGARs and the mean cumulative S-1 dose at 3M and 6M prior to the onset of corneal or lacrimal gland disorders. Our findings suggest that systemic S-1 administration decreases the MGAR in a dose-dependent manner, thus clinicians should pay more attention to morphologic changes to the meibomian glands during early treatment with S-1.

## Appendix

TS-1 Multicenter Study Group: Yuichi Ohashi, Atsushi Shiraishi, Jou Sakai, Yasushi Inoue, Yoshitsugu Inoue, Tsugihisa Sasaki, Taiichirou Chikama, and Masakazu Yamada, Japanese Society of Lacrimal Passage and Tear Dynamics, Japan, Tokyo.
